# Mechanical Properties Study of Miniature Steel Specimens Based on the Small Punch Test and Simulation Methods

**DOI:** 10.3390/ma15196542

**Published:** 2022-09-21

**Authors:** Jingwei Zhang, Zijian Guo, Kanglin Liu

**Affiliations:** College of Chemical Engineering, Fuzhou University, Fuzhou 350108, China

**Keywords:** small punch test, finite element simulation, material properties, compliance calibration, specimen size

## Abstract

The small punch test (SPT) can be very convenient to obtain mechanical properties due to its unique advantages from small-volume samples, and has gained wide popularity and appreciation among researchers. In this paper, the SPT test and finite element (FE) simulations were performed for three alloys, and the yield stresses (*σ_YS_*) and ultimate tensile strengths (*σ_UTS_*) from the uniaxial tensile test (UTT) were correlated with the yield force (*F_y_*) and maximum force (*F_m_*) of the small punch test (SPT) before and after compliance calibration. Finally, the effect of specimen size on the SPT curves was discussed. The results showed that the deviation between SPT test and FE simulation was due to the loading system stiffness, which was confirmed by the loading system compliance calibration test. The SPT curves before and after calibration have less influence on the empirical correlation results for *σ_UTS_*, while the correlation results for *σ_YS_* depend on the method used to determine *F_y_* in the SPT curve. Finally, the simulation results indicated that the effect of specimen size on the force–displacement curve in the SPT is slight. This work also provides a reference for subsequent researchers to conduct empirical correlation studies using different specimen sizes.

## 1. Introduction

In the field of nuclear and petrochemical industries, a large number of facilities are affected by high-temperature environments, neutron irradiation, corrosion, and other harsh environments. After a period of service, the material properties of the equipment will deteriorate. How to obtain real-time mechanical properties of materials without damaging the structural integrity of in-service components has become the focus of many researchers.

As one of the small sample testing techniques (SSTT), the small punch test (SPT) has attracted extensive attention in the field of nuclear and petrochemical industry due to its unique advantages in obtaining material mechanical properties from small volume samples [1,2,3,4,5,6,7,8,9,10]. The SPT method, which originated in the early 1980s, was originally developed to test the changes in material properties caused by tempering embrittlement or irradiation embrittlement of in-service nuclear materials [1]. Now it has gradually developed to evaluate the mechanical property parameters of materials such as tensile properties [2,3,4,5], brittle–ductile transition temperature [6], fracture toughness [7], fatigue property [8,9,10], and creep properties [11,12,13]. In addition, the SPT method is also used to investigate creep crack propagation by some researchers [14,15,16]. The SPT test method is also used in the field of biomechanics [17]. At the same time, because of its advantages in the characteristics of “micro-damage” and “tests”, SPT is particularly useful for some parts that cannot be tested by standardized tests (such as welded joints [18], thermal barrier coatings [19] and functionally gradient materials [20]).

However, despite the many advantages of SPT mentioned above, it faces many problems and challenges nowadays. One of these important issues is how to ensure the accuracy, reliability and comparability of SPT data from different laboratories. Although SPT is widely developed in the last decades, the standardization of this method is still in progress [21,22,23,24,25]. An important factor affecting the results that need to be considered is the accuracy of the correlation results between SPT and the uniaxial tensile test (UTT), which is highly dependent on the sensitivity of the test equipment [26,27,28]. This result makes it difficult to transfer the procedures related to the evaluation of mechanical parameters (*σ_YS_* and *σ_UTS_*) by different researchers [29,30].

To solve this problem, a number of researchers have extensively discussed the different experimental parameters that affect the reliability of SPT results. Lucas et al. [31] first studied the effects of specimen thickness, hole diameter of the lower die, and punch size on force–displacement curves with different materials by experiments. Campitelli et al. [32] studied the effects of specimen thickness and friction coefficient on the force–displacement curves of AISI 316L- and F82H-MOD-tempered martensitic steels by numerical simulation. Xu et al. [33] used the FE method to discuss the influence of the punch diameter and hardness, hole diameter of lower die and chamfer radius, distance of punch from the center and specimen thickness on SPT results. Zhou et al. [34] studied the effects of friction coefficient, specimen thickness, stamping rate and a lower die aperture on force–displacement curves of SUS304 stainless steel by the GTN model; Andrés et al. [35] studied the influence of different clamping conditions on the upper die on the SPT and the small punch creep test (SPC). Peng et al. [36] systematically studied the influence of small deviations of various test parameters on the results of SPT, from the aspects of specimen geometric deviation, material mechanical properties, damage parameters, pre-tightening condition of the upper die and the friction coefficient between the punch or die and the specimen. And finally, the sensitivity of each test parameter in the five stages of the force–displacement curve was summarized. 

However, in addition to the factors mentioned above, there is an important factor whose influence is often ignored, and this is the way in which the value of the displacement used for representing the force-displacement response is determined [37,38]. Different researchers have used different SPT displacement measurement systems, resulting in differences in loading system compliance, which in turn may cause their measured displacement values to shift in the direction of displacement between the SPT test and the FE simulation [39]. This deviation may be more obvious for some non-contact displacement sensors or COD-type extensometers that measure the displacement through the punch in the experimental equipment [40]. Moreno et al. [39] performed a thorough analysis in order to clarify this matter and the results showed that the different force–displacement curves in SPT can be obtained by the above different measurement methods. Hähner et al. [40] also pointed out that the initial stage curves of force–displacement in SPT are affected by using the non-contact displacement transducers. The results of Ávila et al. [27] showed that this factor leads to a deviation between SPT tests and FE simulations, and attributed this uncertainty to the large effect of elastic displacement associated with the low stiffness of the device setup. Although the LVDT sensor is recommended to obtain relatively accurate displacement results through direct contact with the bottom of the specimen in the current standard [21], this displacement measurement system is not used by all researchers. Therefore, it is a matter of discussion whether the direct use of the SPT curve before compliance calibration to correlate it with the material properties produces significantly different results from the empirical correlation of the SPT curve after calibration. In addition, the diameter of a standard small specimen is usually 8 mm or 10 mm, and the specimen’s shape is either round or square, which depends on different countries and regions. As in European Union, the round specimen of Φ 8 mm × 0.5 mm is adopted. In China, round specimens with Φ 10 mm × 0.5 mm are usually used. In contrast, a 10 mm × 10 mm × 0.5 mm or 8 mm × 8 mm × 0.5 mm square specimen can be conveniently sampled from the sharp impact specimen compared with a round specimen [33]. Therefore, it is necessary to investigate the effect of different size tests on the SPT results.

In this paper, the mechanical properties of 316L, 347L stainless steels, and a new high-entropy alloy, Co_32_Cr_28_Ni_32.94_Al_4.06_Ti_3,_ were investigated by SPT and UTT. The effect of loading system compliance on the empirical correlation results between SPT and UTT was systematically investigated. Finally, the effect of specimen sizes on the SPT curves was investigated by the (3D) FE model. The objective of this paper was to evaluate the mechanical properties of steels for pressure vessels by the SPT method and to discuss the results of the empirical correlation between the characteristic parameters (*F_y_* and *F_m_*) on the force–displacement curves before and after the compliance calibration and the material properties (*σ_YS_* and *σ_UTS_*) of the standardized tests. These findings will surely contribute to the future standardization of SPT and provide a reference for subsequent researchers to conduct empirical correlation studies using different specimen sizes.

## 2. Materials and Methods

### 2.1. Materials

**Figure 1:**
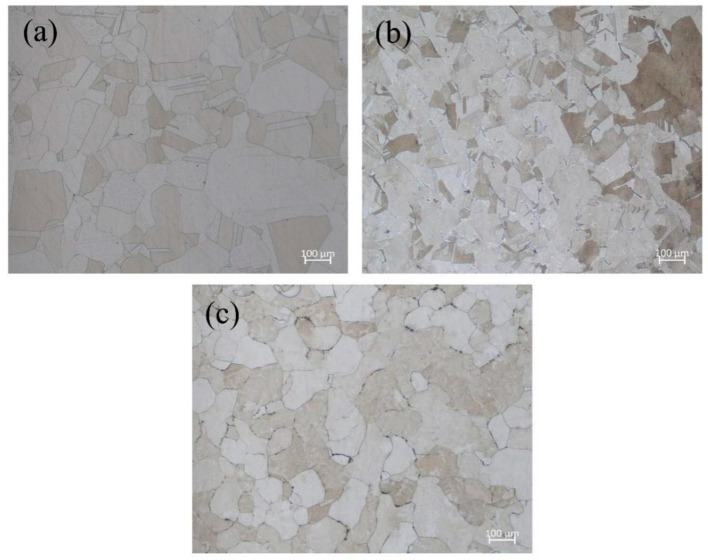
Optical microscope images of the metallographic microstructure of three materials. (**a**) 316L stainless steel; (**b**) 347L stainless steel; (**c**) Co_32_Cr_28_Ni_32.94_Al_4.06_Ti_3_.

The materials used in this paper were 316L, 347L stainless steels and Co_32_Cr_28_Ni_32.94_Al_4.06_Ti_3_ high-entropy alloys, which are generally used for pressure vessels. 316L and 347L stainless steel are widely used in petrochemical and pharmaceutical fields, and Co_32_Cr_28_Ni_32.94_Al_4.06_Ti_3_ is a new high-entropy alloy with promising applications in nuclear power. More details about Co_32_Cr_28_Ni_32.94_Al_4.06_Ti_3_ are introduced in the previous article [41]. The main chemical components were analyzed by EDS (Energy Dispersive Spectroscopy, BRUKER corp., Karlsruhe, Germany). The results are shown in Table 1. The composition (atomic fraction and weight ratio, %) of Co_32_Cr_28_Ni_32.94_Al_4.06_Ti_3_ is shown in Table 2. The optical microscope images of the metallographic microstructure of the above materials are shown in Figure 1. 

### 2.2. Uniaxial Tensile Test

**Figure 2:**
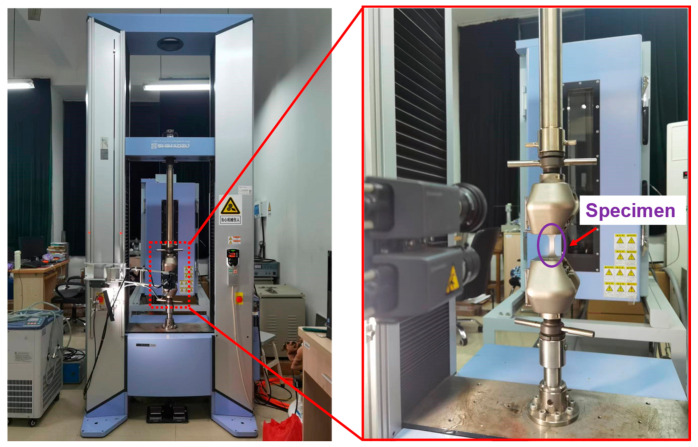
Overview of uniaxial tensile test.

**Figure 3:**
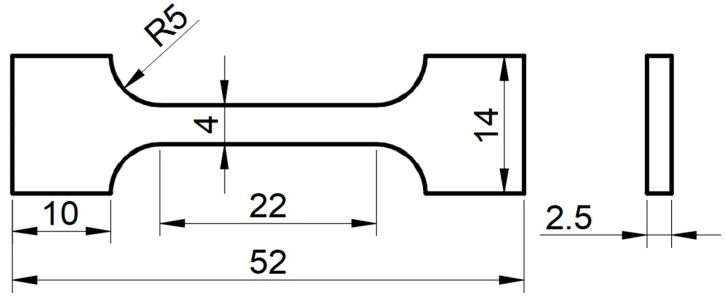
Dimensions of the tensile specimen (mm).

In order to obtain the mechanical properties of the materials, uniaxial tensile tests were carried out at room temperature using AG-X plus a universal electronic testing machine (SHIMADZU corp., Kyoto, Japan), as shown in Figure 2. A 52 mm × 14 mm × 2.5 mm plate-shaped tensile test specimen was used (Figure 3), and tensile tests were carried out at a strain rate of 1 × 10^−3^ mm/s. Finally, the yield stress (*σ_YS_*), ultimate tensile strength (*σ_UTS_*), and uniform elongation (*ε*) of three materials are presented in Table 3.

### 2.3. Small Punch Test

**Figure 4:**
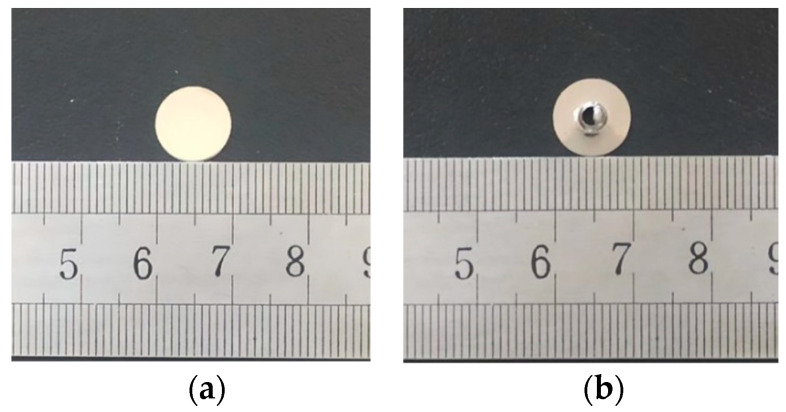
Macro morphology of a small punch specimen. (**a**) before test; (**b**) after test.

SPT specimens with a diameter of 10 mm and a thickness of 0.8 mm were cut from the plate-shaped tensile specimens after the experiment. Then, the specimens were polished to 0.55 mm on both sides by 600, 1200, and 1500 grit size papers, and finally, the specimens were finely polished to a mirror brightness of 0.5 mm by diamond lapping paste to meet the standard requirements for the surface roughness of the specimens. Figure 4 shows the macroscopic morphology of the small punch specimen before and after the experiment.

**Figure 5:**
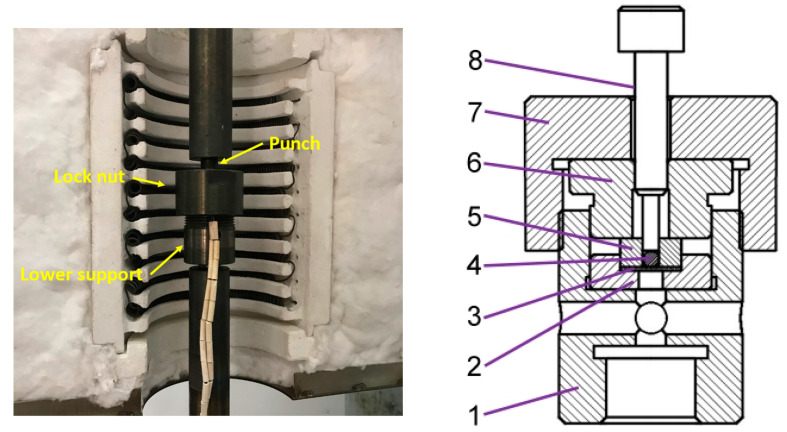
Schematic diagram of small punch test. 1. Lower support 2. Lower die 3. Specimen 4. Ball 5. Guide block 6. Upper die 7. Lock nut 8. Punch.

In this paper, the small punch test apparatus, modified based on the small punch creep test machine, was used to conduct SPT. Its force sensor and displacement sensor are coupled to the loading brake, and the force and displacement changes are recorded by loading the punch. Once the force is received on the force sensor, the displacement sensor begins to record the punch displacement data. This displacement sensor can be used to obtain relatively accurate displacement data of the punch on the top. The schematic diagram of the apparatus is shown in Figure 5. The apparatus for clamping the specimen consists of an upper die, and a circular lower die with a hole diameter of 4 mm. The specimen is placed horizontally and perpendicular to the direction of the force. During the test, the loading is applied to the specimen by means of a punch and a ball with a diameter of 2.5 mm. The test was conducted in a quasi-static condition with a displacement rate of 0.5 mm/min.

### 2.4. FE Model and Numerical Simulation

**Figure 6:**
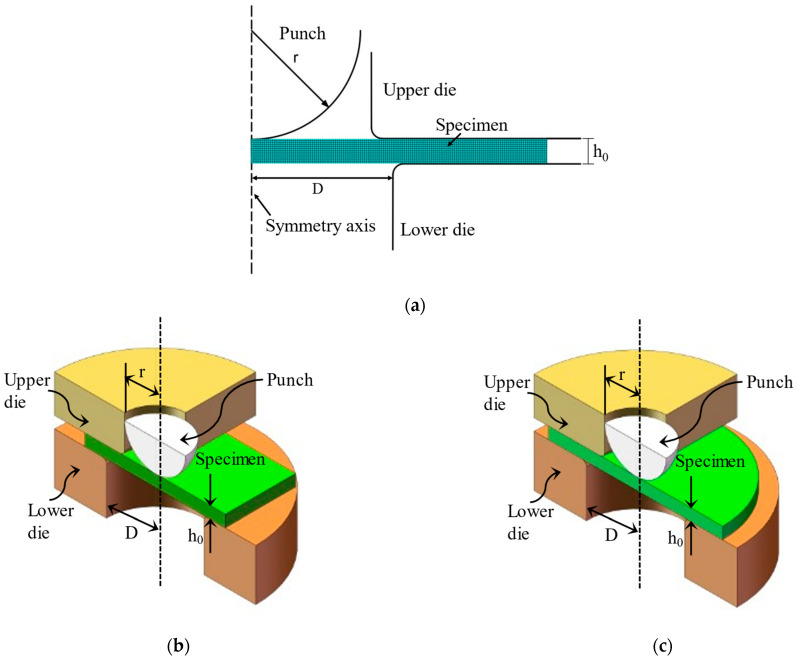
Schematic diagram of FE model of SPT. (**a**) 2D model; (**b**) 3D model with square specimen; (**c**) 3D model with round specimen.

The FE model used in this paper is shown in Figure 6. Both a two-dimensional (2D) axisymmetric model and quarter-symmetric three-dimensional (3D) model were used to simulate the SPT by ABAQUS large commercial FE software [42]. A 2D model with 2000 axisymmetric 4-node square elements, homogenized mesh, and reduced integration with hourglass control (CAX4R) was mainly used to investigate the effect of equipment compliance on experimental data, while a 3D model with about 64,000 8-node linear brick elements, homogenized mesh, and reduced integration with hourglass control (C3D8R) was used to study the effect of specimen size on experimental data. Most researchers widely use these two models in SPT simulations [5,43]. They are suitable for analyzing cases involving large stress and strain gradients, as well as for studying complex contact problems. The agreement between the 2D and 3D model results was confirmed in the article [29,30] by Altstadt et al. Isotropic elastic–plastic materials are subject to the von Mises yield criterion and the corresponding J_2_ flow theory [44,45], which takes the form as follows,

(1) $$2\sigma_{YS}^{2}={\left(\sigma_{1}-\sigma_{2}\right)}^{2}+{\left(\sigma_{2}-\sigma_{3}\right)}^{2}+{\left(\sigma_{3}-\sigma_{1}\right)}^{2}$$

where *σ*_1_, *σ*_2_, and *σ*_3_ are the three principal stresses respectively. The material properties’ inputs in ABAQUS were imported from real stress–strain data evaluated by uniaxial tensile tests of related materials, as shown in Table 3. The constitutive equation can be expressed in the following form,

(2) $$\sigma =\sigma_{YS}+\sigma_{P}\left(\varepsilon_{p}\right)$$

where the *σ_p_*(*ε_p_*) equation is derived from the uniaxial tensile behavior [32]. Generally, the FE model constructed consists of four parts, namely the (1) punch, (2) upper die, (3) lower die, and (4) specimen. Unlike the test, the FE model perfectly simulates the test setup under ideal conditions. The upper and lower dies and punch are defined as rigid bodies, and the specimen is defined as a deformable body. The upper and lower dies were completely fixed to simulate the clamping condition during the experiment, while the displacement constraint was applied to the punch until the specimen fails. The friction coefficient between the punch and specimen was set to 0.2, which is a typical friction coefficient for steel-steel contact in the absence of lubrication [46]. The equipment dimensions for the SPT test and FE simulation were kept consistent. The relevant parameters of specimens and apparatus for different models are summarized in Table 4. The mesh refinement was performed to ensure the accuracy of simulation results. The mesh sizes of square and round specimens in the 3D model were consistent to avoid the impact of mesh sensitivity on simulation results [47,48].

## 3. Results

### 3.1. SPT Experiment Results

**Figure 7:**
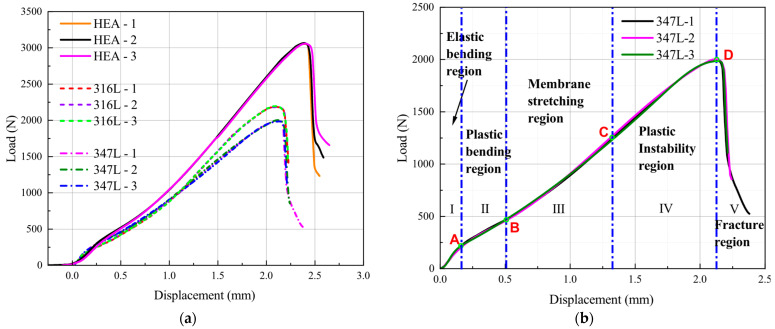
Force–displacement curve of SPT. (**a**) The three materials; (**b**) representation of the different stages.

The typical force–displacement responses of the three materials are shown in Figure 7a. Three test groups were analyzed for each material, and the results show a high degree of coincidence of the force–displacement curves. The force–displacement data of the SPT experiment curve was recorded until the force exceeded the maximum force point and after the specimen ruptured.

As shown in Figure 7b, The force–displacement curve can be divided into five different regions: elastic bending (region I), plastic bending (region II), membrane-stretching (region III), plastic instability (region IV), and unstable fracture (region V). However, the boundaries between these five regions are not clearly defined, and the delineation of the different regions is highly artificial. The force in the initial elastic stage (region I) and plastic stage (region II) increases almost linearly, and an obvious inflection point can be seen between the two regions. This inflection point is called the elastic–plastic transition point. The ordinate value corresponding to this point is defined as the elastic–plastic transition force or yield force (*F_P_*) (corresponding to point A in Figure 7b) by most researchers, which is usually used to correlate with the yield strength (*R_P_*) and regarded as the dividing point between region I and region II. The plastic bending region (region II) ends when the slope of the force–displacement curve increases significantly. In region III, it can be seen that the slope of the force–displacement curve gradually increases. The reason is that after the plastic bending stage, the effect of strain hardening in the material overcomes the reduction of the specimen thickness caused by the punch and enables it to bear the force at an increased rate. This situation is similar to the strain-strengthening stage of the uniaxial tensile test, and the difference is that the stress condition is a biaxial stress state. Therefore, the dividing point between region II and region III (corresponding to point B in Figure 7b) is regarded as the specimen entering the membrane-stretching stage from bending deformation.

The dividing point (corresponding to point C in Figure 7b) between membrane stretching (region III) and plastic instability (region IV) can be regarded as a balance point between the material force bearing and deformation resisting. When the value of force exceeds the value of Point C, due to the decrease in the local thickness of the specimen, the microvoids in the material continuously nucleate, grow, and coalesce, thus inducing microcracks on the specimen surface. On the macro level, the specimen will become softened, and the slope of the force–displacement curve gradually decreases until the maximum force (corresponding to point D in Figure 7b) is reached and the stage of plastic instability (region IV) ends.

**Figure 8:**
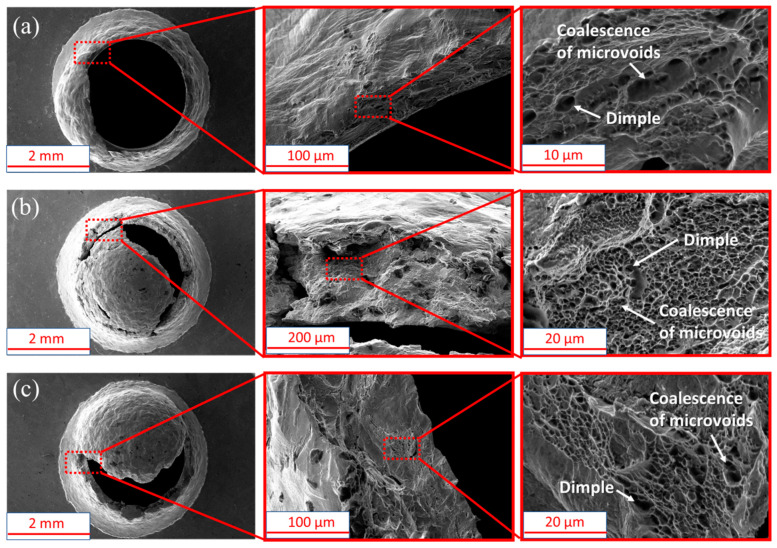
SEM image for three materials. (**a**) 316L stainless steel; (**b**) 347L stainless steel; (**c**) Co_32_Cr_28_Ni_32.94_Al_4.06_Ti_3_.

Figure 8 shows the SEM images of the three materials observed after the specimens fractured (region V). It can be observed that a large number of dimples formed in the material, and a large number of microvoids coalesced. Then, the force of the specimens begins to drop sharply, which is called an unstable fracture region (region V).

### 3.2. SPT Simulation Results

**Figure 9:**
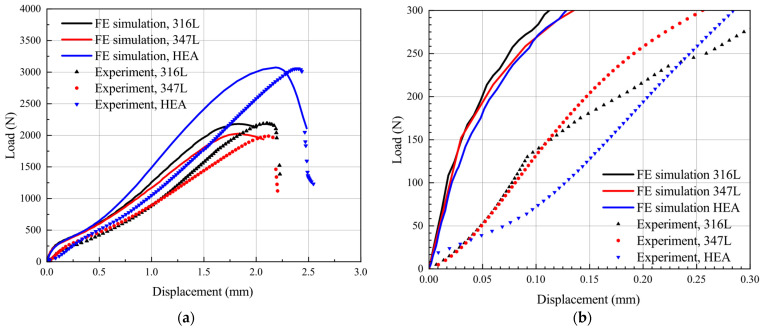
Comparison of force–displacement curve between the experiment and FE simulation. (**a**) at all stages; (**b**) at the initial stage.

Figure 9a shows the experiment and simulation results of the three materials used in this study. When compared with the experiment results, the dividing points in the force–displacement curve of simulation are more pronounced. The general trends of the experiment and simulation responses are approximately similar in the initial four regions but different in the slopes. This deviation is more pronounced at the initial stage of the force–displacement curve, as shown in Figure 9b. The reason can be explained as that, contrary to the experiment, the specimen dies and punch are defined as rigid bodies in the simulation process, but in fact are elastic deformers. In the practical data recording, different displacement measurement methods lead to the displacement sensor at the top of the punch recording additional displacement due to the elastic deformation of the test frame [39] during the loading process, so that the displacement data recorded by the displacement sensor at the top of the punch (corresponding to SPT test) are slightly higher than the displacement data on the upper face of the specimen (corresponding to SPT simulation). The above situation leads to deviations of the force–displacement curves between the test and simulation. This deviation is also reported in literature [28,32].

### 3.3. Calibration of Loading System Compliance

In the previous section, we analyzed and explained the reasons for the deviations between the experimental and simulation results. However, the reasonableness of the analytical results for the curves in Figure 9 needs to be further investigated by applying the compliance calibration of the loading system to the experimentally obtained force–displacement curves. It is worth noting that regarding the compliance calibration of the loading system, different compliance calibration methods are used by researchers [27,28,32,39,40,46,49]. The displacement (*δ_upper_*) on the upper face of the specimen can be derived from the displacements (*δ_ext_*) through an appropriate correction of the respective elastic compliances involved, referred to as *C_ext_* [28,32,39,40]. Some researchers [27,43,46] believe that a linear calibration of the force–displacement (*δ_ext_*) response can be achieved with the compliance calibration curve obtained by this method. However, other researchers have argued that the effective contact area with the punch, the applied stress, and the strain field obtained during the SPT are neither constant nor uniform and continuously vary during the test. At the same time, as the punch displacement increases, the deformation of the specimen and the effective contact area of the material increases, and the specimen stiffness gradually increases [27]. Thus, the nonlinear calibration for the force–displacement (*δ_ext_*) response should be considered in the actual compliance calibration to obtain the best match between the test and simulation curves. Meanwhile, Ávila et al. [27] also pointed out that if only the initial stiffness is used to correct the original curve, a large overcorrection may be obtained for high displacement values.

**Figure 10:**
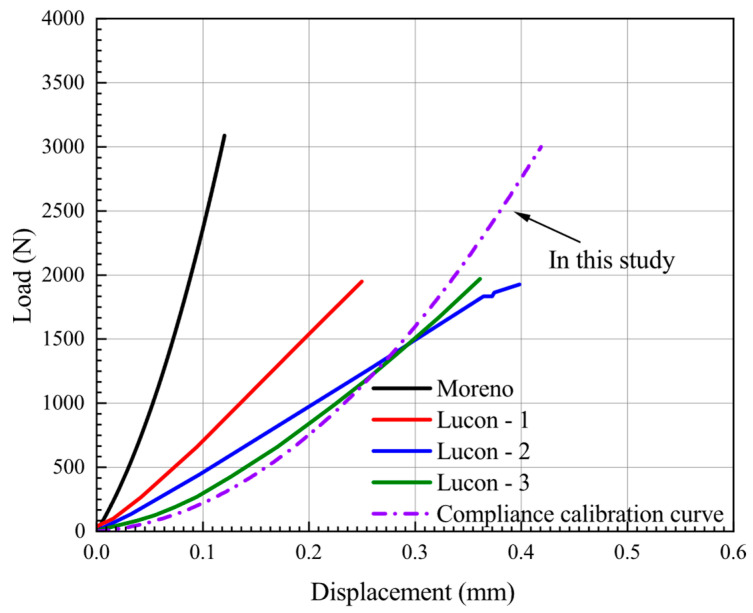
Compliance calibration curve by different researchers.

Therefore, a nonlinear compliance calibration curve was used in this paper. The compliance calibration of the loading system was performed using a cylindrical tungsten carbide specimen with a diameter of 10 mm and a thickness of 5 mm instead of the small circular piece in the test. The calibration method is similar to the reference [43]. In the first loading step, the maximum force was obtained, The subsequent force should not be exceeded in the SPT, and some unloading–loading cycles were performed until a steady state of the force–displacement (*δ_ext_*) curve was reached. The last loading step of this calibration test was recorded, and a fifth-order polynomial regression was established from this data as a calibration equation. This curve was used to calibrate the *δ_ext_* obtained from the SPT test, which resulted in a new displacement (*δ_upper_*) equal to the displacement of the upper surface of the specimen. It should be noted that the high sensitivity of the calibration process on the test apparatus makes the loading–unloading process unstable. Therefore, in addition to the compliance calibration curves obtained in this study, the correction results for AISI 304L by Moreno et al. [39] and the three calibrations for JRQ by Lucon et al. [49] are also shown in Figure 10.

As can be seen from the results in Figure 10, the final calibration curves are different due to the displacement measurement systems, test apparatus, and punch materials used by the researchers. It can also be found that the results in the reference [47] show that there are still slight deviations in the three compliance calibration results for the same material with the same test apparatus. Therefore, the compliance calibrations in Appendix A of EN 10,371 [21] are given as a recommended range.

**Figure 11:**
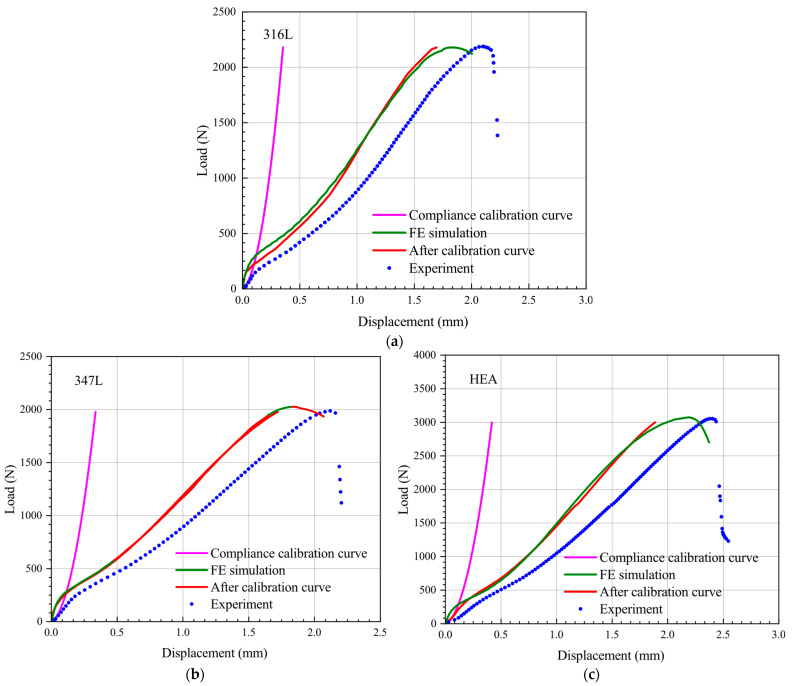
Force–displacement curve of SPT before and after compliance correction. (**a**) 316L; (**b**) 347L; (**c**) HEA.

The compliance curve in Figure 10 can be expressed in the following form:

*δ_Ν_^WC^* = *P*_1_*(F)* (3)

where *δ_Ν_^WC^* is the elastic deformation of the test apparatus and punch, *F* is the corresponding force. The force–displacement curve in Figure 7 can also be expressed in the following form:

*δ_exp_* = *P*_2_*(F)* (4)

where *δ_exp_* includes the elastic bending deformation due to specimen deflection plastic indentation of the punch and elastic deformation of the test frame. Thus, the actual displacement of the specimen can be expressed as:

*δ_upper_* = *δ_exp_* − *δ_Ν_^WC^* = *P*_2_*(F)* − *P*_1_*(F)* (5)

where *δ_upper_* is the displacement of the upper face of the specimen; the corrected results for the above three materials by Equation (5) are shown in Figure 11. As can be seen, the corrected curves obtained using the above calibration method generally agree well with the simulated curves, with only slight deviations at the initial stages of the curves. Therefore, the deviation between the experiment and simulation results can be attributed to the influence of loading system compliance.

## 4. Discussion

### 4.1. Correlation between SPT Curve and Tensile Properties before and after Compliance Calibration

The above results confirm that the deviation between experimental and FE results in the displacement is caused by the compliance of the testing machine configuration and the displacement measurement method. It is important to note that due to the high sensitivity of the SPT, the disturbance generated in the process of correcting the compliance of the loading system may lead to the failure of the calibration process, and extra care should be taken in this process. In fact, due to the different displacement measurement methods and materials in the SPT equipment, the results of the compliance calibration are very different. One of the best methods to avoid this problem is to use LVDT contact displacement measurements directly. This method can directly obtain accurate displacement data at the bottom of the specimen and prevent the possible failure of compliance calibration. However, due to the conditions in different laboratories, direct contact displacement measurements sometimes cannot be used. Thus, the effect of testing machine configuration compliance on the empirical correlation results is worth discussing. 

#### 4.1.1. Correlation with Ultimate Tensile Strength (*σ_UTS_*)

Researchers widely use the maximum force (*F_m_*) as the characteristic force associated with the ultimate tensile strength (*σ_UTS_*). The primary empirical correlation forms are as follows [50]:

(6) $$\sigma_{UTS}=\alpha_{1}\cdot \frac{F_{m}}{h_{0}^{2}}+\alpha_{2}$$

(7) $$\sigma_{UTS}=\alpha_{1}^{\prime }\cdot \frac{F_{m}}{\left(h_{0}\cdot u_{m}\right)}+\alpha_{2}^{\prime }$$

where *h*_0_ is the specimen’s initial thickness, *F_m_* is the maximum force used to determine the force characteristic value of *σ_UTS_*, and *u_m_* is the value of displacement corresponding to the force characteristic *F_m_*. α_1_, α_2_, α_1_’, and α_2_’ are the correlation coefficients related to the material. The results before and after compliance calibration for the three materials are presented in Table 5. 

Equations (6) and (7), respectively, were used to empirically correlate the maximum force (*F_m_*) and the ultimate tensile strength (*σ_UTS_*) of the three materials before and after compliance calibration. Equations (8) and (9) are the empirical correlation equations before compliance calibration and Equations (10) and (11) are the after-compliance calibration equations. The relation between the SPT characteristic force (*F_m_*) and UTT strength property (*σ_UTS_*) can then be expressed as follows: 

Before compliance calibration:

(8) $$\sigma_{UTS}=0.10\cdot \frac{F_{m}}{h_{0}^{2}}-282,R^{2}=0.99$$

(9) $$\sigma_{UTS}=0.67\cdot \frac{F_{m}}{\left(h_{0}\cdot u_{m}\right)}-727,R^{2}=0.99$$

After compliance calibration:

(10) $$\sigma_{UTS}=0.10\cdot \frac{F_{m}}{h_{0}^{2}}-727,R^{2}=0.99$$

(11) $$\sigma_{UTS}=0.72\cdot \frac{F_{m}}{\left(h_{0}\cdot u_{m}\right)}-1090,R^{2}=0.99$$

Comparing Equation (8) with Equation (10), it can be seen that the correlation coefficients in the empirical correlation equations obtained from Equation (6) are less affected before and after the compliance calibration and differ only in the correlation coefficient *α*_2_. However, it can be seen from the results of the compared Equation (9) with Equation (11) that the correlation coefficients are slightly different before and after compliance calibration. The reason for the above situation is that empirical correlation equation, Equation (6), for each material is only concerned with *F_m_*, which hardly changes before and after the calibration, and thus the difference is not significant. For the same reason, the empirical correlation equation, Equation (7), for each material is not only relevant to *F_m_* but to *u_m_*, and *u_m_* changed significantly before and after the calibration, so the data obtained after the compliance calibration of Equation (7) are different from before. Suppose the uncorrected curve is used to evaluate the material ultimate tensile strength (*σ_UTS_*); an overestimation of results may be obtained (i.e., the larger value of *σ_UTS_* will be obtained by the uncorrected curve compared with the corrected curve with the same *F_m_/*(*h*_0_*·u_m_*)), and therefore unsafe results will be obtained.

#### 4.1.2. Correlation with Yield Stress (*σ_YS_*)

**Figure 12:**
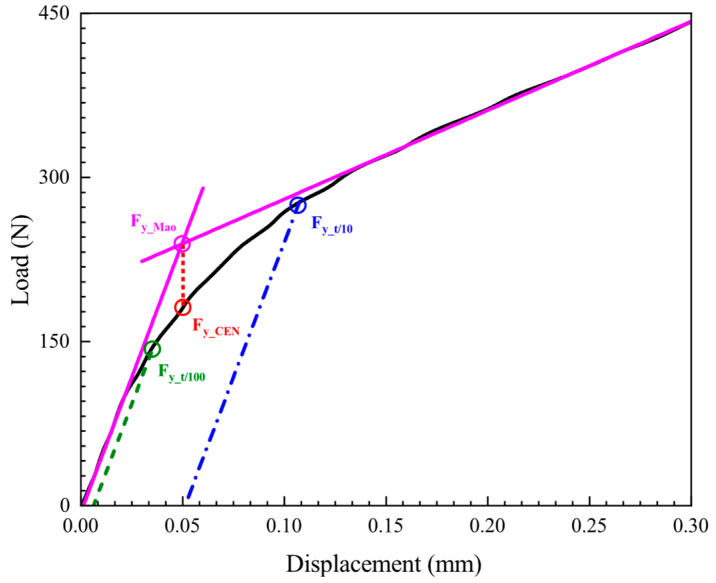
Different methods of determining the force *F_y_* on SPT curve.

The empirical correlation equation of yield stress is usually expressed in the following form,

(12) $$\sigma_{YS}=\beta_{1}\cdot \frac{F_{y}}{h_{0}^{2}}+\beta_{2}$$

where *β*_1_ and *β*_2_ are the empirical correlation constants, *h*_0_ is the initial thickness of SPT specimen, and *F_y_* is the elastic–plastic transition force in the SPT curve. The key to determining yield stress (*σ_YS_*) lies in how to determine the corresponding characteristic force, i.e., elastic–plastic transition force (*F_y_*). Different methods for determining *F_y_* are proposed, as described in references [28,50]. In all the current assessments, *F_y_Mao_* [51], *F_y_CEN_* [22], *F_y_t/10_* [52], and *F*_*y_t*/100_ [6] methods are adopted by most researchers, as shown in Figure 12.

The *Mao* method [51] minimizes the error between the linear function and the SPT initial stage curve by establishing two linear procedures, and the resulting intersection point is *F_y_ Mao,_* and the intersection point projected vertically onto the SPT curve is *F_y_CEN_*. The *t*/10 and *t*/100 methods were performed by drawing a parallel line tangent to the elastic region I of the SPT curve. The line was translated along the deflection axis to the points whose values are *t*/10 and *t*/100, respectively. The intersection value between the parallel line tangent and SPT curve was determined as the characteristic force *F_y_*. The maximum slope of the tangent line corresponding to the slope at the inflection point is suggested in Hähner et al. [40] as the slope of the elastic region I of the SPT curve before compliance calibration, and this method was used in this paper to determine the yield stress (*σ_YS_*) before compliance calibration.

The characteristic forces (*F_y_*) associated with the yield stress (*σ_YS_*) before and after the compliance calibration are listed in Table 6. The relation between the SPT characteristic force (*F_y_*) and UTT material property (*σ_YS_*) can then be expressed as follows: 

Before compliance calibration:

(13) $$\sigma_{YS}=0.36\cdot \frac{F_{y\_Mao}}{h_{0}^{2}}+41.58,R^{2}=0.87$$

(14) $$\sigma_{YS}=0.35\cdot \frac{F_{y\_CEN}}{h_{0}^{2}}+84.80,R^{2}=0.89$$

(15) $$\sigma_{YS}=0.22\cdot \frac{F_{y\_t/10}}{h_{0}^{2}}+134.07,R^{2}=0.73$$

(16) $$\sigma_{YS}=0.31\cdot \frac{F_{y\_t/100}}{h_{0}^{2}}+137.23,R^{2}=0.82$$

After compliance calibration

(17) $$\sigma_{YS}=0.34\cdot \frac{F_{y\_Mao}}{h_{0}^{2}}+56.83,R^{2}=0.85$$

(18) $$\sigma_{YS}=0.32\cdot \frac{F_{y\_CEN}}{h_{0}^{2}}+97.23,R^{2}=0.86$$

(19) $$\sigma_{YS}=0.25\cdot \frac{F_{y\_t/10}}{h_{0}^{2}}+171.74,R^{2}=0.76$$

(20) $$\sigma_{UTS}=0.10\cdot \frac{F_{y\_t/100}}{h_{0}^{2}}-282,R^{2}=0.85$$

where Equations (13)–(16) as well as Equations (17)–(20) correspond to the four methods for determining *F_y_*, respectively. A comparison of the empirical correlation coefficients in the equations above intuitively shows that the differences before and after the compliance correction are obvious for different methods of determining the yield force (*F_y_*). The empirical correlation equations before and after compliance calibration determined using *F_y_Mao_*, *F_y_CEN_*, and *F*_*y_t*/10_ methods showed small deviations, and the empirical correlation coefficients *β*_1_ and *β*_2_ do not differ significantly. In contrast, the empirical correlation equations determined by the *F*_*y_t*/100_ method showed larger deviations in the empirical correlation coefficients *β*_1_ and *β*_2_ before and after compliance calibration, which indicates that the *F_y_Mao_*, *F_y_CEN_*, and *F*_*y_t*/10_ methods are less affected by the compliance calibration and are more applicable to the characteristic forces of the above-mentioned study materials.

It can also be seen that the regression coefficient *R*^2^ of the empirical correlation equation for yield strength (*σ_YS_*) is relatively small compared to the empirical correlation equation for ultimate tensile strength (*σ_UTS_*), indicating that its empirical correlation equation has a large scattering, which may be caused by the small number of data points. Table 6 illustrates the values of the empirical correlation constant *β*_1_ from the literature of different researchers. It should be noted that for the empirical correlation equation of yield strength, the coefficient *β*_2_ may not necessarily exist, depending on the differences in the mechanical properties of the material and the influence of the test apparatus. Table 7 combined with Equations (17) and (18) shows that the values of *β*_1_ determined by the *Mao* and *CEN* methods are close to the values in the literature [5,32,41,42,43,44,45,46,47,48,49,50,51,52,53]. Therefore, by combining the two perspectives above, *F_y_Mao_* and *F_y_CEN_* may be the more desirable characteristic forces for the three materials of interest in this study.

**Figure 13:**
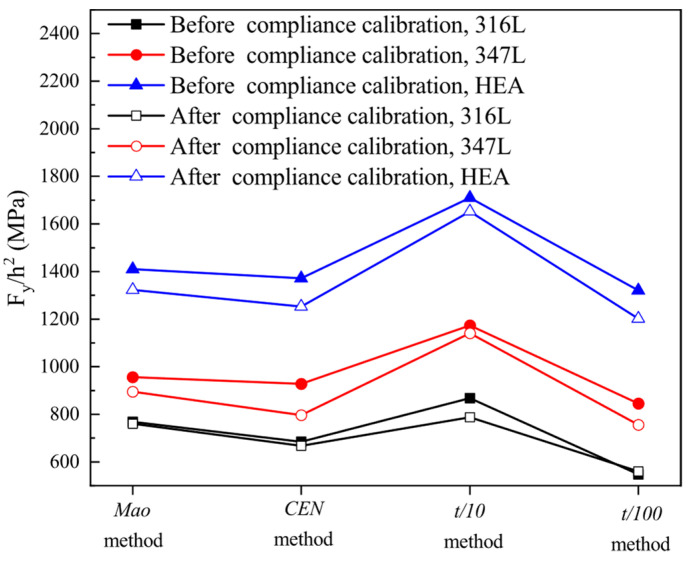
Different methods for determining the *F_y_*.

Figure 13 compares the different methods of determining the yield force (*F_y_*) for the three materials studied in this paper. It can be seen that the difference in the methods of determining the yield force (*F_y_*) before and after compliance correction is slight. This result indicates that the determination of the yield stress (*σ_YS_*) is not affected by the loading system stiffness. It can also be seen that if the empirical correlation coefficient in Equation (8) is constant, the *t*/10 method obtains the highest yield strength of the material, while the other three methods obtain the yield strength of the material relatively close. However, this result was demonstrated only for the three materials studied in this paper, and its applicability to other materials remains further confirmed.

### 4.2. Effect of Different Specimen Sizes on SPT Curve

In order to avoid the influence of some neglected factors on the SPT results during the experiments, the subsequent studies of different specimen sizes were based on FE analysis. The different specimen sizes generally used by most researchers are presented in Table 8. The data of 316L stainless steel mentioned above were used for FE simulations. The mesh sizes of square specimens and round specimens in the 3D model were identical to avoid the impact of mesh sensitivity on simulation results.

**Figure 14:**
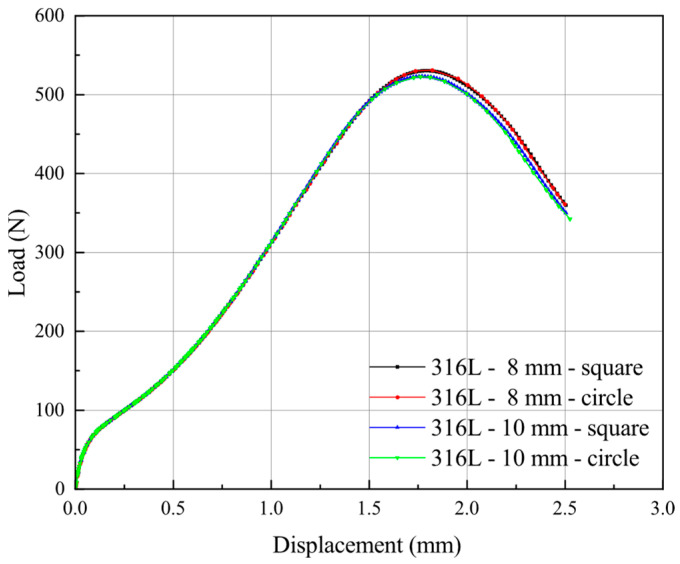
Force–displacement curve of SPT test for specimens in Table 7.

Figure 14 shows the results of the SPT curves for all cases in Table 7. It can be seen that the FE simulation results for round and square specimens with the same diameter exhibit almost identical SPT curves. This result shows that the SPT curves were not influenced by the specimen’s shape when the specimen periphery was in a fully clamped condition. Moreover, for specimens with diameters of 8 mm and 10 mm, respectively, there is a high degree of coincidence in the first four regions of the SPT curves. Still, the two curves begin to diverge near the maximum force, and the general downward trend in force remains consistent beyond the maximum force. In general, the effect of specimens’ diameter on the force–displacement curve is not apparent, and a slight difference induced by the specimen diameter can be found beyond the maximum force point.

**Figure 15:**
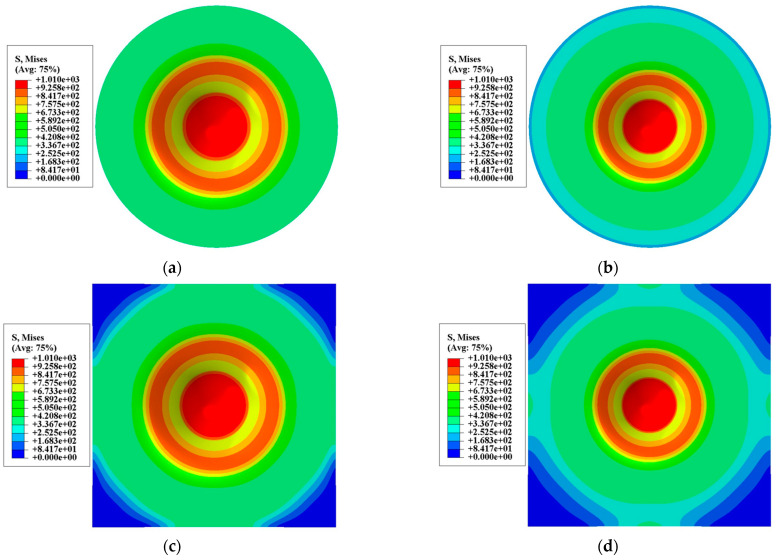
Contour plots for Von Mises stress on FE model of SPT. (**a**) round specimen with a diameter of 8 mm; (**b**) round specimen with a diameter of 10 mm; (**c**) square specimen with a diameter of 8 mm; (**d**) square specimen with a diameter of 10 mm.

Figure 15 shows the equivalent stress distribution of four different types of specimens of 316L stainless steel when the punch displacement was 1.7 mm. It can be seen from the figure that although the overall stress distribution of square specimens and round specimens is different, the stress distribution of the contact part with the punch at the center of the specimen is roughly the same. The difference between round or square specimens with different diameters is only evident in the non-contact area with the punch.

**Figure 16:**
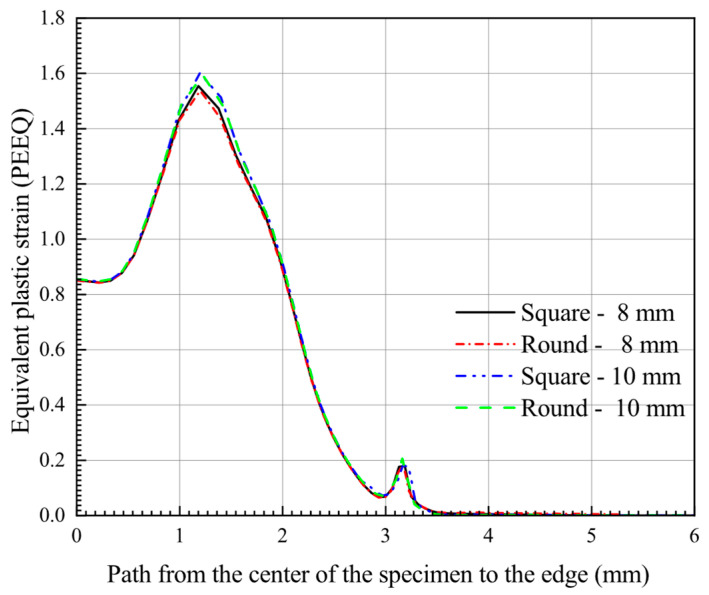
PEEQ distribution on the bottom surface of the specimen at the maximum force in the path from the center of the specimen to the edge.

Figure 16 shows the equivalent plastic strain distribution on the bottom surface of the specimen at the maximum force in the path from the center of the specimen to the edge. Combined with the contour plots of the equivalent plastic strain distribution for specimens of different diameters and shapes in Figure 15, it can be more intuitively seen that the different specimen sizes have almost the same equivalent plastic strain distributions for the SPT deformation process.

Based on the above results, it can be seen that different specimen sizes, i.e., round and square specimens with different diameters kept clamped around, have little effect on the SPT curve results. This implies that the results obtained are theoretically transferable and comparable when SPT tests are performed by different researchers using square and circular specimens, respectively. However, this conclusion must be carried out under the condition that the periphery of the specimen is wholly clamped in the experimental results.

## 5. Conclusions

In this paper, SPT experiments and simulations were carried out on 316L, 347L stainless steels, and a new high-entropy alloy Co_32_Cr_28_Ni_32.94_Al_4.06_Ti_3_, and the effect of specimen sizes on the SPT curve were investigated by simulation. The main conclusions are as follows:

(1) The discrepancy of the force–displacement of SPT between the test and the simulation results is mainly due to the loading system stiffness.

(2) The empirical correlation results for the ultimate tensile strength (*σ_UTS_*) by Equation (6) and for the yield strength (*σ_YS_*) by Equation (12) have little difference before and after the loading system compliance calibration, but the correlation results by Equation (7) have a greater difference because of the changes in *u_m_* before and after the loading system compliance calibration.

(3) Simulation results on different specimen shapes and sizes show that the effect of specimen shapes and sizes on the SPT results can be ignored. This conclusion was confirmed by the 316L stainless steel simulation results. Therefore, the experimental results obtained based on the above specimen size provide a reference for different researchers even if their specimen shapes and sizes differ. 

This investigation shows that insufficient stiffness of a loading system can lead to an inaccurate material properties by the small punch test, but the results after correction are still trustworthy. It also provides a reference for subsequent researchers to conduct empirical correlation studies using different specimen sizes.

## Figures and Tables

**Table 1:** Chemical composition of stainless steel (wt%).

Steel	Chemical Composition (wt%)								
	C	Si	Mn	P	S	Cr	Mo	Ni	Nb
316L	0.015	0.70	0.54	0.02	0.007	16.53	2	11.55	—
347L	0.04	0.75	1.97	0.02	0.01	18.5	—	11.0	0.95

**Table 2:** Atomic and weight ratios of the principal elements of Co_32_Cr_28_Ni_32.94_Al_4.06_Ti_3_.

Composition	Co	Cr	Ni	Al	Ti
Atomic ratio (%)	32	28	32.94	4.06	3
Weight ratio (%)	34.11	26.34	34.97	1.98	2.60

**Table 3:** Mechanical properties of three materials at room temperature.

Materials	Yield Stress (*σ_YS_*)/MPa	Ultimate Tensile Strength (*σ_UTS_*)/MPa	Uniform Elongation (*ε*)/%
316L	350	635	58.1
347L	320	540	9.5
Co_32_Cr_28_Ni_32.94_Al_4.06_Ti_3_	530	985	37.2

**Table 4:** Geometric parameters of SPT for 2D and 3D models.

Set No.	Specimen Type	Specimen Diameter d (mm)	Punch Radius r (mm)	Receiving Hole Radius D (mm)	Specimen Thickness h_0_ (mm)	Edge Type
2D	round	10 mm	1.25 mm	4 mm	0.5 mm	Full clamped
3D	round	10 mm/8 mm	1.25 mm	4 mm	0.5 mm	Full clamped
3D	square	10 mm/8 mm	1.25 mm	4 mm	0.5 mm	Full clamped

**Table 5:** Characteristic parameters (maximum force) of the SPT curve before and after compliance calibration.

Compliance	Steel	*u_m_* (mm)	*F_m_*/*h*_0_^2^ (MPa)	*F_m_*/(*h*_0_·*u_m_*) (Mpa)
Before	316L	1.70	8720	2565
	347L	2.14	8000	1869
	Co_32_Cr_28_Ni_32.94_Al_4.06_Ti_3_	2.40	12,212	2544
After	316L	2.10	8748	2573
	347L	1.83	8088	2573
	Co_32_Cr_28_Ni_32.94_Al_4.06_Ti_3_	2.19	12,268	2800

**Table 6:** Characteristic force of the SPT curve determined by different methods before and after compliance calibration (yield force).

Compliance	Steel	*F_y_Mao_/h*_0_^2^ (MPa)	*F_y_CEN_/h*_0_^2^ (MPa)	*F_y_t/10_/h*_0_^2^ (MPa)	*F*_*y_t*/100_/h_0_^2^ (MPa)
Before	316L	768	684	868	548
	347L	956	928	1173	845
	Co_32_Cr_28_Ni_32.94_Al_4.06_Ti_3_	1410	1372	1710	1321
After	316L	760	668	788	560
	347L	895	797	1141	756
	Co_32_Cr_28_Ni_32.94_Al_4.06_Ti_3_	1323	1253	1652	1202

**Table 7:** Recommended values of parameters *β*_1_ and *β*_2_ in different literature.

*β*_1_	*β*_2_	Materials	Method	Reference
0.36	-	SUS316, PCA, HT-60, A533B, HT-9	*F_y_Mao_*	Mao and Takahashi [51]
0.39	-	316L and F82H	*F_y_CEN_*	Campitelli et al. [32]
0.38	-	HAZ of 30CrMo5-2	*F_y_CEN_*	Rodriguez et al. [52]
0.364	-	Different steels and Al alloy	*F*_*y_t*/10_	Garcia et al. [50]
0.442	-	Different steels and Al alloy	*F_y_CEN_*	Garcia et al. [50]
0.476	-	Different steels and Al alloy	*F_y_Mao_*	Garcia et al. [50]
0.349	133.48	Ti-6Al-4V, Stainless Steel, Cu, Al, In718	*F_y_Mao_*	Lancaster et al. [53]

**Table 8:** FE model parameters of 316L stainless steel with different specimen sizes.

Material	Specimen Diameter (mm)		Specimen Shape	
316L	8	10	Round specimen	Square specimen

## Data Availability

Not applicable.
